# State-of-the-art treatment of metastatic renal cell carcinoma

**DOI:** 10.3747/co.v16i0.407

**Published:** 2009-05

**Authors:** D.Y.C. Heng, C. Kollmannsberger

**Keywords:** Renal cell carcinoma, vascular endothelial growth factor, platelet-derived growth factor, mammalian target of rapamycin inhibitors, sunitinib, sorafenib, temsirolimus

## Abstract

Targeted therapy has greatly changed the way in which metastatic renal cell carcinoma (rcc) is treated. Agents that inhibit the vascular endothelial growth factor and mammalian target of rapamycin pathways that otherwise lead to angiogenesis have now become the standard of care. Much research into the sequence and combination of these agents is ongoing, and new anti-angiogenic agents are being developed. This overview covers the standard treatment of metastatic rcc with targeted therapy, immunotherapy, and surgery. Future directions and ongoing clinical trials are also discussed.

## 1. INTRODUCTION

Metastatic renal cell carcinoma (rcc) is estimated to have caused 13,010 deaths in the United States in 2008 1. Previously, immunotherapy agents such as interleukin-2 and interferon alpha (ifnα) were the only treatments available, and they demonstrated low response rates of approximately 15% 2–7. The biology underlying rcc has been elucidated, and agents targeting relevant biologic pathways have been investigated 8. This targeting developed chiefly from an understanding of von Hippel–Lindau (vhl) syndrome, which is an inherited, autosomal dominant genetic disorder that commonly manifests in the development of clear-cell rcc in affected patients. The present review details the current evidence behind traditional approaches, including cytoreductive nephrectomy, metastasectomy, and immunotherapy, and then provides an overview of the contemporary targeted therapies and related ongoing clinical trials.

## 2. SURGERY IN PATIENTS WITH METASTATIC DISEASE

Radical nephrectomy has been indicated even for rcc patients with metastatic disease. Two phase iii studies randomized patients to ifnα with or without cytoreductive radical nephrectomy. In a combined analysis of these trials, median survival was 7.8 months in patients treated with ifnα alone as compared with 13.6 months for patients who received ifnα plus cytoreductive nephrectomy 9–11. A greater survival advantage was observed in patients with better performance status. This cytoreductive paradigm remains predominant in the era of targeted therapy because clinical trials demonstrating the benefits of targeted therapy have largely been observed in the context of prior nephrectomy 12–16. It remains to be seen whether the observation of shrinkage in the primary tumour with targeted agents may alter this paradigm in future. A randomized trial to clarify the value of cytoreductive nephrectomy is currently in development (A. Ravaud, personal communication).

Although cytoreductive nephrectomy appears to benefit many patients with metastatic rcc, it is not curative, and it should not be performed indiscriminately. Patients who are most likely to benefit from cytoreduction include those with

- substantial tumour burden (for example, in excess of 75%) in the involved kidney,
- good performance status, and
- no central nervous system or liver metastases (with rare exceptions) 17.

Other considerations pertain to surgical resectability, particularly the potential for morbidity if proximity to vital structures, encasement of the renal hilum, or other complicating factors are present. Because of lower operative morbidity and mortality, laparoscopic nephrectomy is emerging as the standard surgical procedure whenever technically feasible.

Patients with disseminated rcc and a solitary metastasis may be considered for metastasectomy, although they represent a small fraction of cases. Favourable prognostic factors include a long interval between initial diagnosis and development of the metastasis, which reflects an indolent course and reinforces the likelihood that the metastasis is truly solitary. Additionally, complete resection of the metastatic disease should be possible (for example, solitary pulmonary nodule), so that the patient can be said to have no evidence of disease 18. Patients with favourable-to-intermediate prognostic features may achieve a 5-year survival of 38%–71% with metastasectomy, and thus surgical resection of metastases should be considered in appropriately selected rcc patients 19.

## 3. IMMUNOTHERAPY

Early trials using chemotherapy did not produce a significant benefit 20,21, and so, in an attempt to harness the innate immune response of rcc tumours, immunotherapy has long been the standard of care for the treatment of metastatic rcc. Treatment with ifnα produced response rates of up to 15%, with modest to no prolongation of overall survival (os), in comparisons with inactive controls 5,6. A pooled analysis investigating the use of high-dose interleukin-2 (il-2) revealed an overall response rate (orr) of 14%, with 5% complete responses 22. Most patients achieving a complete response had durable disease remission. Notably, significant side effects were observed, including capillary leak syndrome, which necessitated intensive blood pressure monitoring and the occasional requirement for vasopressors.

Phase iii trials of high-dose il-2 failed to demonstrate significant benefit in comparisons with low-dose cytokine regimens 2,3. High-dose il-2 is noteworthy for a small but real percentage of durable complete remissions; however, this treatment strategy is able to be applied only in a small, highly selected fraction of rcc cases. Even the occasional patient with very extensive disease, including involvement of lung, bone, or liver, may sustain a remission measured in years. Attempts to precisely characterize and identify this group of responders have been uniformly unsuccessful, however, which makes the use of this treatment strategy very challenging.

## 4. BIOLOGIC BASIS OF TARGETED THERAPY: VHL

The recognition of hereditary renal neoplasms catalyzed the discovery of the genetic basis of rcc. Clinically, the vhl syndrome, a constellation of cysts and tumours in the central nervous system and abdominal viscera 8, is inherited in an autosomal dominant fashion. The central nervous system lesions include retinal hemangioblastomas, endolymphatic sac tumours, and craniospinal hemangioblastomas. The visceral lesions in these patients include clear-cell rccs, pheochromocytomas, neuroendocrine pancreatic tumours, epididymal cystadenomas, and broad ligament cystadenomas.

Patients with vhl syndrome have an aberrant *VHL* allele on chromosome 3p25, which predisposes them to disease if the second allele is mutated. This configuration is a prime example of the classical “two-hit hypothesis” for genes with a tumour-suppressor function. The vhl 213-amino-acid protein polyubiquinates hypoxia-inducible factor (hif), which marks that factor for destruction by the cellular proteasome. Normally, low oxygen conditions allow hifα to accumulate and bind to hifβ, thereby creating a complex that transcriptionally activates genes. In patients with aberrant *VHL*, hifα is left to freely accumulate without degradation, even under normal oxygen conditions. Thus, the transcription of genes related to glucose metabolism, apoptosis, angiogenesis, and endothelial stabilization are abnormally promoted. This disordered response to hypoxia activates more than 100 hif-responsive genes that include growth factors and their receptors such as vascular endothelial growth factor (vegf), platelet-derived growth factor (pdgf), and transforming growth factors alpha and beta 23.

Nonhereditary, sporadic clear-cell rcc also exhibits *VHL* aberrations 23. A single *VHL* allele deletion occurs in approximately 78.4%–98% of sporadic tumours 24–29. For the remaining allele, *VHL* gene mutations are seen in 34%–57%, and gene inactivation via hypermethylation of CpG–rich dna islands occurs in about 5%–20.4% of clear-cell rcc 24,27,28,30,31. Thus, it is clear that, in hereditary and sporadic cases of clear-cell rcc alike, *VHL* abnormalities are a key in the pathogenesis.

## 5. TARGETED THERAPY

With an understanding of the biology behind metastatic rcc, new drugs have been developed to target downstream effectors of vhl and hif (Table i), including vegf, pdgf, and mammalian target of rapamycin (mtor). Drugs such as sunitinib and sorafenib target the vegf receptor (vegfr), and drugs such as bevacizumab target the vegf ligand. Other drugs such as temsirolimus and everolimus target downstream effectors of vegf, including mtor kinase. All of these drugs have demonstrated efficacy in the treatment of metastatic rcc and have been incorporated into the current treatment algorithm (Table ii).

### 5.1 VEGFR Tyrosine Kinase Inhibitors

Receptor tyrosine kinases (rtks) play an integral role in the signalling cascade of vegf and pdgf 34. An extracellular domain of the rtks binds to the respective ligand, and an intracellular domain holds the tyrosine kinase responsible for downstream signalling. Upon ligand binding, the rtks dimerize or multimerize to induce a conformational change that permits binding of adenosine triphosphate, resulting in autophosphorylation and transphosphorylation. These tyrosine domains are then able to phosphorylate and activate various proteins in the downstream signal transduction cascade.

#### 5.1.1 Sunitinib

Sunitinib is an oral multikinase inhibitor that blocks vegfrs 1, 2, and 3, pdgf receptor beta and related rtks 35. Initial phase ii trials of sunitinib in metastatic rcc (in 169 patients who had failed prior cytokine-based therapy) demonstrated an investigator-assessed objective response rate of 45%, a median duration of response of 11.9 months, and a median progression-free survival (pfs) of 8.4 months 36–38.

The pivotal phase iii randomized controlled trial (750 patients) compared first-line sunitinib with ifnα and demonstrated a statistically significant advantage in objective response rate (39% vs. 8%, *p* < 0.000001) and pfs (11 months vs. 5 months) with a hazard ratio (hr) of 0.42 (*p* < 0.001) 12. The median os of the sunitinib and interferon groups was 26.4 months and 21.8 months respectively, which was of borderline statistical significance (*p* = 0.051), likely because patients who progressed on ifnα were allowed to cross over to receive vegf-targeted therapy 32. Notably, most of the enrolled patients (94%) had a favourable or intermediate risk by Memorial Sloan–Kettering Cancer Center (mskcc) prognostic criteria 39. Common toxicities included fatigue, hand–foot syndrome, diarrhea, mucositis, hypertension, and hypothyroidism. Cardiotoxicity has been reported, and thus monitoring may be required in patients with pre-existing heart disease 40.

Because of these phase ii and iii trials, sunitinib has become a standard of care for the first-line treatment of metastatic rcc.

#### 5.1.2 Sorafenib

Sorafenib was initially investigated for its ability to inhibit the Raf protein kinase, thereby affecting the mitogen-activated protein kinase signalling pathway responsible for downstream proliferation responses. However, it subsequently became clear that activity against vegfr 2, vegfr 3, pdgf beta, Flt-3, and c-Kit was also present 41.

The Treatment Approaches in Renal Cancer Global Evaluation Trial, the largest study of previously-treated metastatic clear-cell rcc, enrolled 903 patients who were randomized either to oral sorafenib 400 mg twice daily or placebo 13. All patients enrolled had favourable or intermediate risk mskcc prognostic criteria. A clear median pfs benefit (5.5 months vs. 2.8 months) was observed in the sorafenib group. Objective response rate was minimal (10%, investigator-assessed), although more than 70% of patients had some degree of tumour burden reduction. The toxicities commonly experienced with sorafenib are similar to those experienced with sunitinib, except that the hand–foot syndrome may be more pronounced, and cardiotoxicity appears to occur less frequently.

Based on these data, sorafenib has been approved by the U.S. Food and Drug Administration (fda) and has become a standard of care for second-line treatment of metastatic rcc after immunotherapy failure. However, in a randomized phase ii trial of first-line sorafenib versus ifnα, a pfs benefit could not be demonstrated 42. Thus, sorafenib has assumed a largely second-line or later role in the treatment of metastatic rcc. The exception is the first-line use of sorafenib in patients unsuitable for sunitinib.

### 5.2 VEGF Ligand–Directed Therapy

Bevacizumab is a recombinant monoclonal antibody that binds and neutralizes circulating vegf. The activity of this agent in rcc was initially identified by small randomized trials 43,44. A subsequent phase iii clinical trial randomized nephrectomized patients with clear-cell metastatic rcc to the combination of ifnα (three times weekly at a dose of 9×10^6^ IU for up to 1 year) plus bevacizumab [10 mg/kg intravenously (IV) every 2 weeks], or ifnα with placebo until disease progression 14. The addition of bevacizumab to ifnα significantly increased pfs (10.2 months vs. 5.4 months; hr: 0.63; *p* < 0.0001) and the objective tumour response rate (31% vs. 13%, *p* < 0.0001). A trend toward improved os was observed with the addition of bevacizumab, although results are not yet mature (*p* = 0.0670). A Cancer and Leukemia Group B phase iii trial of similar design confirmed a pfs (8.5 months vs. 5.2 months, *p* < 0.0001) and an orr (25% vs. 13%, *p* < 0.0001) benefit with bevacizumab plus ifnα 33. The final analysis of os is pending. Common toxicities included hypertension and proteinuria, with rare but serious toxicity including bowel perforation, arterial ischemic events, and bleeding.

Whether bevacizumab must be given in combination with interferon is unknown, because no bevacizumab monotherapy arm was used in the phase iii trials. Nevertheless, bevacizumab is important in the armamentarium of treatments for rcc and fda approval is pending.

### 5.3 mTOR Inhibitors

Another downstream effect of the vegfr pathway is activation of Akt and phosphoinositide 3 kinase, which in turn promote mtor kinase. A component of intracellular pathways, mtor promotes tumour growth and proliferation, and is a mediator of the hypoxic response.

#### 5.3.1 Temsirolimus

Temsirolimus is an fda-approved mtor inhibitor that binds to FK506 binding protein 1A to create a complex that directly inhibits mtor. A phase iii trial included 626 previously untreated patients with poor prognostic criteria and randomized them to temsirolimus 25 mg IV weekly, ifnα 18×10^6^ IU 3 times weekly, or temsirolimus plus ifnα 3 times weekly 16. Patients were required to have 3 or more of the following adverse risk features:

- Karnofsky performance status below 80
- Lactate dehydrogenase more than 1.5 times the upper limit of normal
- Hemoglobin below the lower limit of normal
- Serum corrected calcium above 10 mg/dL
- Less than 1 year from first diagnosis of rcc to start of therapy
- Three or more metastatic sites

Of the patients included in this trial, 19% had non-clear-cell or unknown histology. Temsirolimus monotherapy demonstrated an os advantage in comparison with infα (10.9 months vs. 7.3 months, log-rank *p* < 0.008). The orr was 8.6% for temsirolimus and 4.8% for interferon, which was not a statistically significant difference. Common side effects included fatigue, hypercholesterolemia, and hyperglycemia. Temsirolimus has become a first-line standard of care for patients with metastatic rcc, appropriately applied to patients with poor prognostic criteria.

#### 5.3.2 Everolimus

Another mtor inhibitor, everolimus (RAD001), has recently been reported in a phase iii trial to improve the pfs of patients with metastatic rcc who progressed on sunitinib, sorafenib, or both 45. These patients were randomized to daily oral everolimus 10 mg or placebo and were stratified by number of previous tyrosine kinase inhibitors (tkis) and mskcc “previously treated” risk groups (1 point each for anemia, hypercalcemia, and Karnofsky performance status below 80, with 0 points = favourable risk, 1 point = intermediate risk, and 2+ points = poor risk). The primary endpoint was pfs, and in the RAD001 and placebo groups, that endpoint was 4.0 months and 1.9 months respectively (*p* < 0.0001). For the two groups, the os was not reached and 8.8 months respectively (*p* = 0.233, but the analysis is not yet mature). The benefit in pfs was seen in all three mskcc risk groups. Common side effects included asthenia, anemia, and stomatitis. This is the first second-line trial after initial tki failure to demonstrate benefit. Regulatory approval is pending.

## 6. DRUGS IN DEVELOPMENT

Axitinib (AG013736) is a small-molecule tki of vegfr, pdgf receptor, and c-Kit. A phase ii trial enrolled 62 treatment-refractory patients with rcc that had progressed on sorafenib 46. They were treated with oral axitinib 5 mg twice daily. Of 62 patients, 13 exhibited a partial response, and the median pfs was 7.4 months. An earlier phase ii trial enrolled cytokine-refractory nephrectomized patients who demonstrated a response rate of 44.2% and a median time to progression of 15.7 months with axitinib 47. Currently, a large multicentre phase iii trial is enrolling patients that progressed on sunitinib and is randomizing them to axitinib or sorafenib (search for “NCT00678392” at clinicaltrials.gov/ct2/search).

Pazopanib (GW786034) is another tki of vegfrs 1–3, pdgf receptors alpha and beta, and c-Kit. A randomized discontinuation study was initiated in patients who were treatment naïve (68%) or who had one line of immunotherapy (25%), bevacizumab (3%), or other non-targeted therapy (2%). The first 60 patients demonstrated good disease control rates, leading the Data and Safety Monitoring Committee to stop the discontinuation randomization phase and to allow all patients to continue the drug. Among the 225 patients with metastatic rcc enrolled, an orr of 27% was observed by independent review at 12 weeks 48. A first-line pazopanib versus sunitinib randomized controlled trial is currently recruiting (search for “NCT00720941” at clinicaltrials.gov/ct2/search).

Cediranib (AZD2171) is an potent inhibitor of vegfrs 1–3, pdgf receptor beta, and Flt-4. This oral agent has been studied by the Princess Margaret Hospital Consortium in a phase ii trial as first-line treatment in patients with progressive, unresectable, advanced metastatic rcc 49. Preliminary results indicate a partial response rate of 38% (6/16 patients). An additional 6 patients had stable disease, and 3 patients had progressive disease. Further trials examining this agent are warranted.

Volociximab is a chimeric monoclonal antibody against α5β1 integrin. The antibody blocks fibronectin in the extracellular matrix from binding to α5β1 integrin, inducing apoptosis of proliferating endothelial cells. Volociximab was studied in a multicentre phase ii study in patients with metastatic clear-cell rcc that enrolled 40 evaluable patients. It was well tolerated at 10 mg/kg given IV every 2 weeks. One subject achieved a partial response, and 32 subjects had stable disease 50.

Inhibitors of c-Met such as XL880 and ARQ197 have been developed with the knowledge that genetic alterations in papillary rcc are different from those in clear-cell rcc. In patients with heritable disease, the c-met proto-oncogene on chromosome 7 is frequently duplicated 51. XL880 and ARQ197 are inhibitors of the c-Met rtk, which is mutated in most heritable papillary cancers and in some sporadic papillary cancers. Dose-finding phase i studies have been completed 52,53, and these novel agents are currently being studied in patients with metastatic papillary rcc in phase ii trials (search for “NCT00345423” at clinical trials.gov/ct2/search). Preliminary results for XL880 (an inhibitor of c-Met and vegfr 2) have been reported in a phase ii trial that enrolled 20 evaluable patients: 3 of those patients had partial responses, and none had progressive disease 54. Of the patients enrolled in the phase i ARQ197 trial, 5 had advanced rcc. Of those 5 patients, 3 had stable disease, 1 had progressive disease, and 1 has yet to be reported 53.

## 7. FUTURE DIRECTIONS

Targeted therapies have now replaced the relatively ineffective complement of immunotherapeutic agents used in patients with metastatic rcc. Whether targeted therapies are best given in sequence or in combination, and what their role in the perioperative setting might be, is currently unclear.

A phase ii trial has completed enrolment of patients who progressed on sunitinib or bevacizumab and who are now receiving sorafenib based on the rationale that sorafenib may have non-cross-resistant activity in these patients. Of the 37 patients enrolled, 52% have experienced tumour shrinkage (defined as a 5% or greater decrease in tumour measurements), and 14% have experienced a true partial response by the Response Evaluation Criteria in Solid Tumors 55. These findings demonstrate that patients that have progressed on one vegf inhibitor can still respond to another vegf inhibitor. However, the effects of these second-line strategies on pfs or os are currently unclear and can be determined only in randomized trials, which are ongoing. At the present time, it remains unknown whether a patient who progressed on a vegf inhibitor should then be exposed to another tki or should be treated using an agent with a different mechanism of action (for example, the mtor inhibitor temsirolimus). A phase iii randomized trial in patients refractory to sunitinib is currently comparing temsirolimus with sorafenib (search for “NCT00474786” at clinicaltrials.gov/ct2/search).

Studies combining targeted therapies are being performed with the known caveat that combination therapies are associated with high financial cost and possibly increased toxicity because of overlapping side-effect profiles. A phase i trial of bevacizumab and sunitinib in a variety of solid tumours (led by the Cleveland Clinic) reported 1 unconfirmed partial response in a patient with papillary rcc from among 9 evaluable patients 56. Another phase i trial of this combination, given exclusively to patients with metastatic rcc, reported partial responses in 4 of 13 patients 57. A randomized phase ii trial studying the combination of bevacizumab and erlotinib [an inhibitor of the epidermal growth factor receptor (egfr) pathway] as compared with bevacizumab and placebo revealed no benefit for the active combination in terms of orr or pfs 44. Currently, combinations of targeted therapy remain experimental, and they should be administered only in the context of a clinical trial.

Targeted agents are also being studied in the adjuvant setting for patients with resected high-risk rcc and in the neoadjuvant setting in an attempt to downstage tumours before surgical resection of localized disease. The Adjuvant Sorafenib or Sunitinib for Unfavorable Renal Carcinoma Intergroup trial randomizes high-risk nephrectomized patients to one year of sorafenib, sunitinib, or placebo. Other trials such as the Sunitinib Trial in Advanced Renal Cancer (search for “NCT00326898” at clinicaltrials.gov/ct2/search) and the sorafenib versus placebo trial in patients with resected intermediate-or high-risk rcc (search for “NCT00492258” at clinicaltrials.gov/ct2/search) will help to elucidate the effect of these agents in the adjuvant setting. In the neoadjuvant setting, sunitinib in two preoperative cycles is being studied in a phase ii trial (search for “NCT00480935” at clinicaltrials.gov/ct2/search).

## 8. CONCLUSIONS

Targeted therapy has changed the landscape of treatment options for metastatic rcc. The disordered response to hypoxia found in rcc has been harnessed, making vegf, pdgf, and mtor-directed pathways a standard of care. This change has led to improved response rates and prolonged survival. New drug development, sequencing and combinations of drugs, and use of targeted drugs in the adjuvant and neoadjuvant settings are the subjects of ongoing clinical trials.

## Figures and Tables

**TABLE I:** Selected clinical trials of targeted agents in metastatic renal cell carcinoma

*Agent*	*Mechanism*	*Population*	*Trial arms*	*Efficacy* *rr* *(%)*	*pfs* *(months)*	*os* *(months)*
Sunitinib 12,32	Tyrosine kinase inhibitor of vegf and related receptors	First line	Sunitinib vs. interferon	39	11	26.4
				8	5	21.8
				*p*<0.000001	*p*<0.001	*p* =0.051
Sorafenib 13	Tyrosine kinase inhibitor of vegf and related receptors	Treatment-refractory, second line	Sorafenib vs. placebo	10	5.5	17.8
				2	2.8	15.2
				*p*<0.001	*p*<0.01	*p*=0.146
Temsirolimus 16	Inhibitor of mtor	Poor-risk first line	Temsirolimus vs. interferon	8.6	na	10.9
				4.8	na	7.3
				ns		*p*<0.008
Bevacizumab 14,33	vegf ligand–binding antibody	First line	Bevacizumab plus interferon vs. placebo plus interferon	31, 25	10.2, 8.5	na
				13, 13	5.4, 5.2	na
				*p*<0.0001	*p*<0.0001	

rr = risk ratio; pfs = progression-free survival; os = overall survival; vegf = vascular endothelial growth factor; mtor = mammalian target of rapamycin; na = not available; ns = nonsignificant.

**TABLE II:** Current treatment algorithm for patients with metastatic renal cell carcinoma

*Setting*	*Patients*	*Therapy (level 1 evidence)*	*Alternatives*
First-line	Good or intermediate risk	Sunitinib Bevacizumab^a^ plus interferon	High-dose interleukin-2 Sorafenib Observation Clinical trial
	Poor risk	Temsirolimus	Sunitinib Clinical trial
Second-line	Cytokine-refractory	Sorafenib	Sunitinib Bevacizumab^a^ plus interferon Clinical trial
	Prior vegf or mtor	Everolimus^a^ Clinical trial	Targeted therapy not previously used Clinical trial

^a^ Everolimus and bevacizumab are not yet approved by the U.S. Food and Drug Administration for metastatic renal cell carcinoma. vegf = vascular endothelial growth factor; mtor = mammalian target of rapamycin.
